# Bacterial urinary tract infections and urogenital schistosomiasis co-infection among primary school children in a Meatu district, Tanzania: A call for an integrated preventive approach

**DOI:** 10.1371/journal.pone.0362097

**Published:** 2026-11-02

**Authors:** Johannes Rukyaa, Prisca Damiano, Vitus Silago, Domenica Morona, Jeremiah Seni, Humphrey D. Mazigo, Stephen E. Mshana

**Affiliations:** 1 Department of Microbiology and Immunology, Weill Bugando School of Medicine, Catholic University of Health and Allied Sciences, Mwanza, Tanzania; 2 Department of Medical Parasitology and Entomology, Weill Bugando School of Medicine, Catholic University of Health and Allied Sciences, Mwanza, Tanzania; George Washington University School of Medicine and Health Sciences, UNITED STATES OF AMERICA

## Abstract

**Background:**

Urogenital schistosomiasis (UGS) remains a significant public health issue in Tanzania, co-infection with urinary tract infections (UTIs) can complicate diagnosis due to overlapping symptoms. Additionally, gastrointestinal carriage of multidrug-resistant bacteria (MDRB) increases risks of self-infection and transmission to others. Primary school-aged children in rural settings are particularly at risk for both diseases due to poor water sanitation and risky behaviors. This study reports the prevalence and patterns of UGS and UTI co-infection, and MDRB gut carriage among primary school-aged children.

**Methodology:**

A school-based cross-sectional study was conducted in Meatu district, Tanzania (Oct–Dec 2024). UGS was diagnosed using urine filtration, UTIs through urine culture while stool samples were screened for Extended spectrum beta lactamase (ESBL) producing bacteria. Descriptive analysis was done using STATA 15.0.

**Results:**

Among 405 children enrolled, the prevalence of UGS, UTIs, and UGS–UTI co-infection was 44.1%, 12.8%, and 5.2%, respectively. Heavy UGS infection was associated with a greater likelihood of UTI than light infection. Haematuria was associated with UGS, whereas frequent and painful urination were common among children with UTIs and co-infection. Escherichia coli was the predominant UTI pathogen, with substantial resistance to third-generation cephalosporins. ESBL-producing bacteria and multidrug-resistant bacteria were also identified among UTI isolates.

**Conclusions:**

Co-infection of UGS & UTI among primary school children is a noticeable problem in the study settings; fever, frequent urination and painful urination are good predictors for co-infection. Similarities in clinical symptoms among co-infected individuals may complicate diagnosis and management on the initial assessment. High proportion of MDRB carriage in rural settings indicates the extensive burden of antimicrobial resistance in developing countries.

## Introduction

Urogenital schistosomiasis (UGS), caused by *Schistosoma haematobium*, is a significant public health concern in sub-Saharan Africa, with school-aged children being the most affected group. In Tanzania, urogenital schistosomiasis (UGS) remains a significant public health problem, particularly among pre-school and primary school children in north-western Tanzania, with a reported prevalence of 7.4% despite years of mass drug administration [1]. The burden of disease is often compounded by co-infection with bacterial urinary tract infections (UTIs), which can worsen clinical outcomes and complicate initial diagnosis and treatment [2,3].

Primary school children in endemic areas are at increased risk for both UGS and bacterial UTIs due to behavioral factors such as frequent contact with contaminated water, swimming in local rivers, streams, dams and poor hygiene practices, these activities expose children to infected snail-infested waters and fecal contamination, increasing the risk of both parasitic and bacterial infections [4,5].

On the other hand UGS contributes to tissue damage through a chronic inflammatory response induced by the deposition of *S. haematobium* eggs in the bladder and ureters, this inflammation leads to ulceration and fibrosis of the urinary tract lining, compromising the mucosal barrier and creating an entry point for secondary bacterial infection [6–8].

A major challenge in managing UGS and bacterial UTI co-infection lies in the similarity of clinical presentations, symptoms such as hematuria, dysuria, and abdominal pain are common to both infections, making it difficult to distinguish between them during initial assessment without laboratory confirmation [9,10]. This diagnostic overlap may result in mismanagement—either under-treating one infection or prescribing unnecessary antibiotics for another, such practices not only delay appropriate care but also contribute to the growing threat of antimicrobial resistance (AMR) [11,12].

Furthermore, the carriage of multidrug-resistant (MDR) bacteria in the gastrointestinal tract adds another layer of complexity. Children colonized with MDR bacteria may develop urinary tract infections through endogenous (auto) infection when these organisms gain access to the urinary tract. Inadequate sanitation and poor hand hygiene in school environments may facilitate the transmission of MDR bacteria among children through contaminated hands and shared sanitation facilities, thereby increasing the reservoir of resistant organisms within the community [13,14]. In the context of already weakened urinary mucosa due to schistosomiasis, MDR bacteria can establish infections that are difficult to treat with standard antibiotics. Therefore, this study aimed to determine the prevalence of bacterial UTI and UGS co-infection, identify associated risk factors, assess antimicrobial resistance patterns of uropathogens, and determine gastrointestinal carriage of MDRB among primary school children in Meatu District, Tanzania.

## Methods

### Study design, area and population

This was a school-based cross-sectional study conducted in Meatu District, Tanzania, between October and December 2024. As no published estimates of the prevalence of UGS–bacterial UTI co-infection were available for the study setting or comparable populations at the time of study design, the sample size was calculated using the previously reported prevalence of urogenital schistosomiasis (17.7%) in the study area [15]. UGS was selected as the reference prevalence because the study was conducted in a UGS-endemic region and aimed to estimate the burden of UGS and its co-occurrence with bacterial UTI. Using the Kish and Leslie formula for cross-sectional studies, the minimum required sample size was 224 participants [16]. However, a total of 411 primary school-aged children (from Class 1 to Class 7) attending three co-education primary schools in Meatu District, Mwanza Region, were enrolled in the study. six students were excluded due to either the inability to provide an adequate urine sample or the absence of a stool sample, resulting in a final sample size of 405 participants.

### Data & sample collection

A structured, pre-tested questionnaire was administered to collect relevant socio-demographic information, including age, sex, and class level, as well as self-reported clinical information, including dysuria (painful urination), haematuria, lower abdominal pain, and increased urinary frequency at the time of enrolment. Body temperature was measured objectively using a calibrated axillary digital thermometer (OMRON Eco Temp Basic; Omron Healthcare Co., Ltd., Kyoto, Japan) to assess the presence of fever. Following the interview and clinical assessment, each participant was verbally instructed by a trained research assistant on the proper procedure for collecting approximately 10 mL of clean-catch midstream and last-voided urine into a sterile, wide-mouthed urine container (Mshale Tanzania Limited, Dar es Salaam, Tanzania). Participants then collected the urine specimens privately in designated toilet facilities to ensure confidentiality throughout the collection process. To minimize the risk of cross-contamination, stool samples were subsequently collected in sterile, wide-mouthed stool containers (Mshale Tanzania Limited, Dar es Salaam, Tanzania).

### Urine culture

Using a 1 μL sterile disposable loop, a portion of the homogenized urine sample was aseptically inoculated onto Blood Agar (BA) and MacConkey Agar (MCA), both obtained from OXOID (Hampshire, United Kingdom). The inoculated plates were incubated aerobically at 36 ± 1 °C for 18–24 hours. A culture was considered to show significant bacterial growth if ≥10 colonies of a single morphological type were observed [17].

### Urine filtration for *S. haematobium*

To detect *Schistosoma haematobium* eggs, 10 mL of each well-homogenized urine sample was drawn using a sterile 10 mL plastic syringe (Neomedic Limited, Leavesden, United Kingdom) and filtered through a 20 μm pore-size polycarbonate membrane filter (Sterlitech, Kent, WA, USA). The retained material on the filter was then stained with Lugol’s iodine to enhance visibility and examined microscopically at 40 × magnification for the presence of *S. haematobium* eggs [18].

### Stool culture

A portion of the fresh stool sample was aseptically collected using a sterile disposable wire loop and inoculated onto MacConkey agar (MCA) plates supplemented with 2 µg/mL of cefotaxime to selectively screen for extended-spectrum beta-lactamase (ESBL)-producing bacteria. The inoculated plates were then incubated at 36 ± 1 °C for 18–24 hours to allow for bacterial growth and identification [19].

### Bacteria identification

Identification of the bacterial pathogens was based on their growth characteristics on blood agar (BA) and MacConkey agar (MCA), followed by Gram staining and in-house biochemical tests. For Gram-negative bacteria, tests such as Christensen urea agar, triple sugar iron (TSI) agar, Simmons citrate agar, sulphur indole motility (SIM) agar, and oxidase were used. For Gram-positive bacteria, catalase, coagulase, bile aesculin, optochin, novobiocin, and DNase tests were performed [20,21].

### Antimicrobial susceptibility testing

A single colony of bacteria from a fresh pure culture plate was emulsified in sterile normal saline to achieve a concentration equivalent to 0.5 McFarland turbidity solution. A sterile cotton swab was then used to obtain bacteria from the suspension, the swab was then be squeezed against the wall of the tube to remove excess fluid before being seeded uniformly onto a Muller-Hinton agar (OXOID, Hampshire, United Kingdom) plate. Antibiotics of the right potency were placed on the agar plate to test for susceptibility patterns using Kirby Bauer’s disc diffusion method as guided by the Clinical Laboratory Standard Institute (CLSI) 30th edition M100 document, 2024 [22,23]. Antibiotic discs used for Gram-negative bacteria included ceftriaxone (30 µg), cefepime (30 µg), ciprofloxacin (5 µg), ceftazidime (30 µg), piperacillin-tazobactam (100/10 µg), amoxicillin/clavulanate (20/10 µg), trimethoprim-sulfamethoxazole (1.25/23.75 µg), meropenem (10 µg), gentamicin (10 µg), nitrofurantoin (300 µg) and tetracycline (30 µg).

For eligible Gram-negative bacteria, ESBL phenotype was confirmed using the combined disc method with 30 µg cefotaxime and/or 30 μg ceftazidime (with and without 10 µg clavulanic acid) [23].

### Quality control

Control strains of *Staphylococcus aureus* ATCC 25923 and *Escherichia coli* ATCC 25922 were used for quality control of the culture media and the antibiotic discs [23]. MDR was confirmed when resistance to three or more classes of antibiotics was observed among the isolated bacterial pathogens [24].

### Statistical analysis

Data were analysed using STATA version 15 (StataCorp LLC, College Station, TX, USA). Descriptive statistics were used to summarize participants’ characteristics. Categorical variables were summarized as frequencies and percentages, while continuous variables were summarized using the median and interquartile range (IQR). Univariate logistic regression was initially performed to assess the association between each independent variable and UGS–UTI co-infection. Variables with a p-value <0.20 in the univariate analysis were entered into a multivariable logistic regression model to identify factors independently associated with UGS–UTI co-infection while adjusting for potential confounding. The multivariable model included age, sex, haematuria, frequent urination, fever, and abdominal pain. Associations were reported as adjusted odds ratios (aORs) with 95% confidence intervals (CIs), and a two-sided p-value <0.05 was considered statistically significant.

### Ethics statement

This study was conducted as part of a larger project that received ethical clearance from the National Health Research Ethics Review Committee (NATHREC), under certification number NIMR/HQ/R.8a/Vol.IX/4667. Prior to data and specimen collection, permission to carry out the study was obtained from the relevant regional and district authorities. Informed voluntary assent was secured from the parents or guardians of all participants. Throughout the study, strict confidentiality of participant information was maintained during and after the study.

## Results

### Socio-demographic and clinical characteristics of the study participants

A total of 405 primary school children from Meatu district were enrolled in this study, with a median age of 11 (IQR: 6–18) years. Females accounted for 56.5% (229/405) of the study participants, and most were in Class 3 (31.9%, 129/405). Fever and frequent urination were significantly associated with urinary tract infection (UTI) (p < 0.001), while hematuria showed a significant association with Urogenital schistosomiasis (UGS) (p < 0.001). Additionally, painful urination was significantly associated with both UTI and urinary schistosomiasis (p < 0.001), as detailed in Table 1.

**Table 1:** Socio demographic and clinical characteristics of the study participants.

Variables	UTI		P value	Schistosomiasis		P value
	YES, N= 52 (%)	NO, N= 353 (%)		YES, N= 180 (%)	NO, N= 225 (%)	
**Age**	11 [IQR 6-18] years					
**Sex**						
Male	26 (50)	150 (42.5)	0.157	85 (47.2)	91 (40.4)	0.172
Female	26 (50)	203 (57.5)		95 (52.8)	134 (59.6)	
**Sampled site**						
School 1	12 (22.6)	43 (12.2)	0.108	25 (13.9)	30 (13.3)	0.180
School 2	24 (45.3)	192 (54.6)		104 (57.8)	112 (49.8)	
School 3	17 (32.1)	117 (33.2)		51 (28.3)	83 (36.9)	
**Class level**						
Class 2	23 (44.2)	98 (27.8)	0.947	49 (27.2)	72 (32.0)	0.619
Class 3	13 (25.0)	116 (32.9)		62 (34.4)	67 (29.8)	
Class 4	12 (23.1)	89 (25.2)		48 (26.7)	53 (23.6)	
Class 5	1 (1.9)	8 (2.3)		3 (1.7)	6 (2.7)	
Class 6	3 (5.8)	42 (11.8)		18 (10.0)	27 (12.0)	
**Abdominal pain**						
Yes	12 (23.1)	80 (22.7)	<0.001	46 (25.6)	46 (20.4)	0.223
No	40 (76.9)	273 (77.3)		134 (74.4)	176 (79.6)	
**Fever**						
Yes	38 (73.1)	100 (28.3)	<0.001	60 (33.3)	78 (34.7)	0.778
No	14 (26.9)	253 (71.7)		120 (66.7)	147 (65.3)	
**Frequent urination**						
Yes	44 (84.6)	150 (42.5)	<0.001	81 (45.0)	113 (50.2)	0.296
No	8 (15.4)	203 (57.5)		99 (55.0)	112 (49.8)	
**Painful urination**						
Yes	42 (80.8)	199 (56.4)	0.182	131 (72.8)	110 (48.9)	<0.001
No	10 (19.2)	154 (43.6)		49 (27.2)	115 (51.1)	
**Hematuria**						
Yes	24 (46.1)	129 (36.5)	0.365	130 (72.2)	23 (10.2)	<0.001
No	28 (53.9)	224 (63.5)		50 (27.8)	202 (89.8)	
**UTI**						
Yes	NA	NA	0.528	21 (11.7)	31 (13.8)	0.528
No				159 (88.3)	194 (86.2)	
**Schistosomiasis**						
Yes	21 (11.7)	31 (13.8)	0.308	NA	NA	NA
No	159 (88.3)	194 (86.2)				

### Prevalence of UTI and UGS

Out of the 405 primary school children enrolled in this study, 12.8% (52/405) had microbiologically confirmed UTI, while 44.4% (180/405) were diagnosed with UGS. Of the 180 children with UGS, 27.2% (49/180) had a heavy infection load (i.e., > 50 *Schistosoma haematobium* eggs/ 10 mls of urine). Notably, 5.2% (21/405) of the children had both UTI and urinary schistosomiasis. UTI was significantly higher in children with heavy *S. haematobium* infection load vs light (34.6% vs 5.8%, p < 0.001). A total of 52 bacterial pathogens causing UTIs were isolated, with *Escherichia coli* being the most prevalent, accounting for 57.6% (30/52) of the isolates, as illustrated in Fig 1.

**Fig 1:**
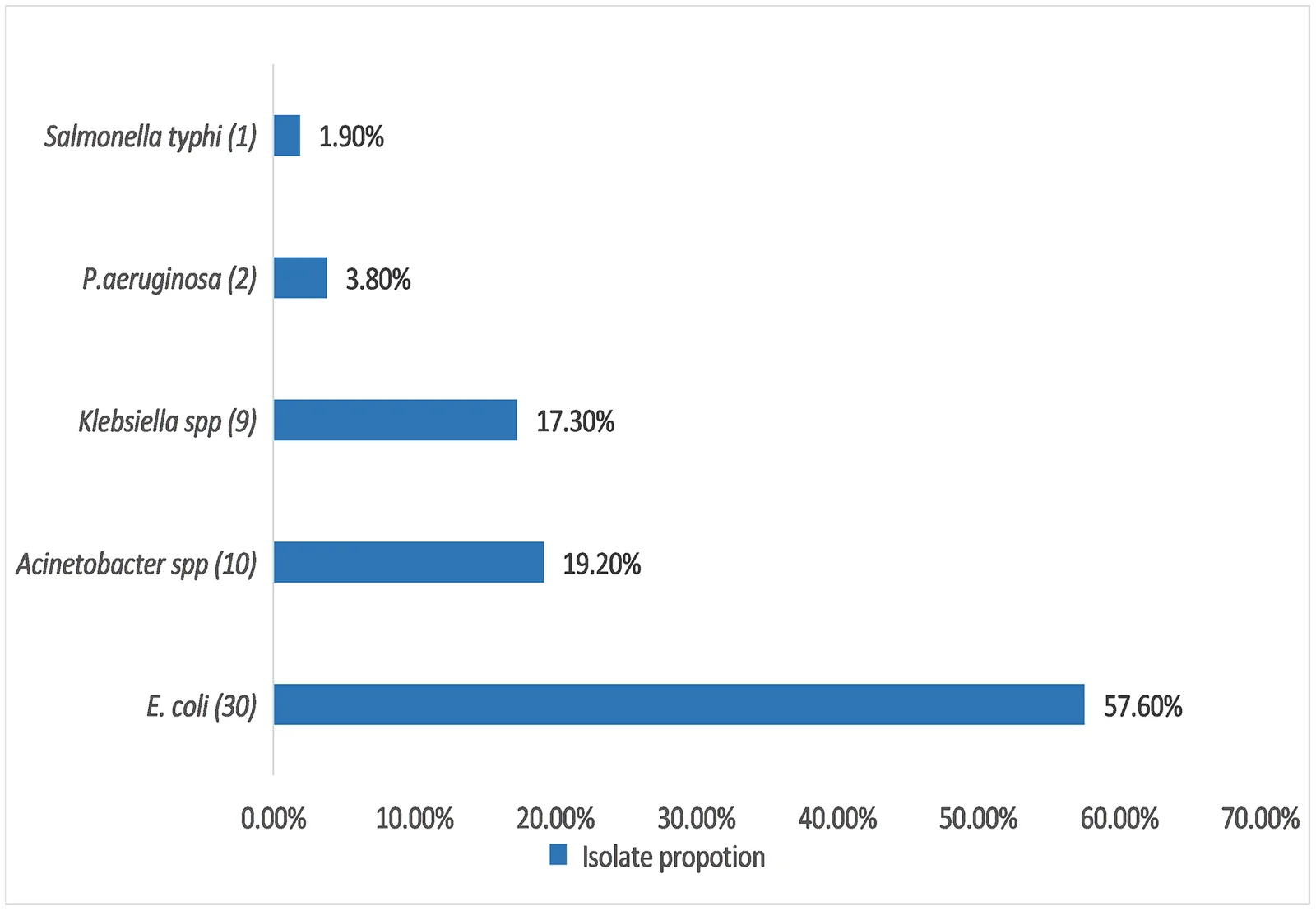
Bacteria pathogens causing Urinary Tract Infection.

### Factors associated with co-infection with UTI and UGS among primary school children

A total of 405 primary school children were included in the analysis, of whom 21 (5.2%) had UTI and urogenital schistosomiasis (UGS) co-infection. In the univariate analysis, fever, frequent urination, painful urination, and hematuria were significantly associated with co-infection (p < 0.001), whereas sex and abdominal pain were not statistically significant. In the multivariate logistic regression analysis, children presenting with fever were significantly more likely to have co-infection compared to those without fever (AOR = 7.58, 95% CI: 2.42–39.00, p < 0.001). Similarly, frequent urination was independently associated with increased odds of co-infection (AOR = 9.71, 95% CI: 2.58–22.23, p = 0.001). Although abdominal pain (AOR = 3.13, 95% CI: 0.97–10.14, p = 0.057) and hematuria (AOR = 3.20, 95% CI: 0.97–10.62, p = 0.057) showed increased odds of co-infection, these associations did not reach statistical significance. Sex was not associated with co-infection (AOR = 0.60, 95% CI: 0.21–1.71, p = 0.340). Painful urination was omitted from the multivariate model due to zero counts in one category as shown in Table 2.

**Table 2:** Logistic analysis for factors associated with UTI and UGS co-infection among primary school children.

	Univariate (Chi test)		Multivariate		
**Patients Variables (N = 405)**	**Co-infection** **N = 21 (%)**	**P value**	**Adjusted OR**	**95% CI**	**P value**
**Sex**					
Male (176)	9 (42.9)	1	1		
Female (229)	12 (57.1)	0.955	0.60	0.211- 1.711	0.340
**Fever**					
No (267)	6 (28.6)	1	1		
Yes (138)	15 (71.4)	<0.001	7.58	2.419-39.00	<0.001
**Abdominal pain**					
No	14 (66.7)	1			
Yes	7 (33.3)	0.233	3.13	0.966-10.143	0.057
**Frequent urination**					
No (210)	3 (14.3)	1			
Yes (195)	18 (85.7)	<0.001	9.71	2.579-22.23	0.001
**Painful urination**					
No (164)	0(0.0)	1	^*^Omitted		
Yes (241)	21(8.7)	<0.001			
**Hematuria**					
No	4 (19.0)	1	1		
Yes	17 (81.0)	<0.001	3.203	0.967- 10.618	0.057

*Logistic analysis for this variable was omitted because it predicts failure perfectly.

### Antimicrobial susceptibility profile of bacterial pathogens causing UTI

Among the 52 isolates bacterial pathogens causing UTI, high resistance proportion was observed towards trimethoprim/sulfamethoxazole 42.0% (21/50) (trimethoprim/sulfamethoxazole *and* tetracycline were not applicable for testing against *Pseudomonas aeruginosa*) and tetracycline 40.0% (20/50) (trimethoprim/sulfamethoxazole *and* tetracycline were not applicable for testing against *Pseudomonas aeruginosa*) respectively. In contrast, low resistance proportion was observed towards meropenem at 5.7% (3/52) as shown in Fig 2. Extended-spectrum beta-lactamase (ESBL) production was detected in 20.0% (8/40) (ESBL testing was not applicable for *Acinetobacter* spp. and *Pseudomonas aeruginosa*) of the eligible Gram-negative bacteria. Additionally, 36.5% (19/52) of all isolates were classified as multidrug-resistant.

**Fig 2:**
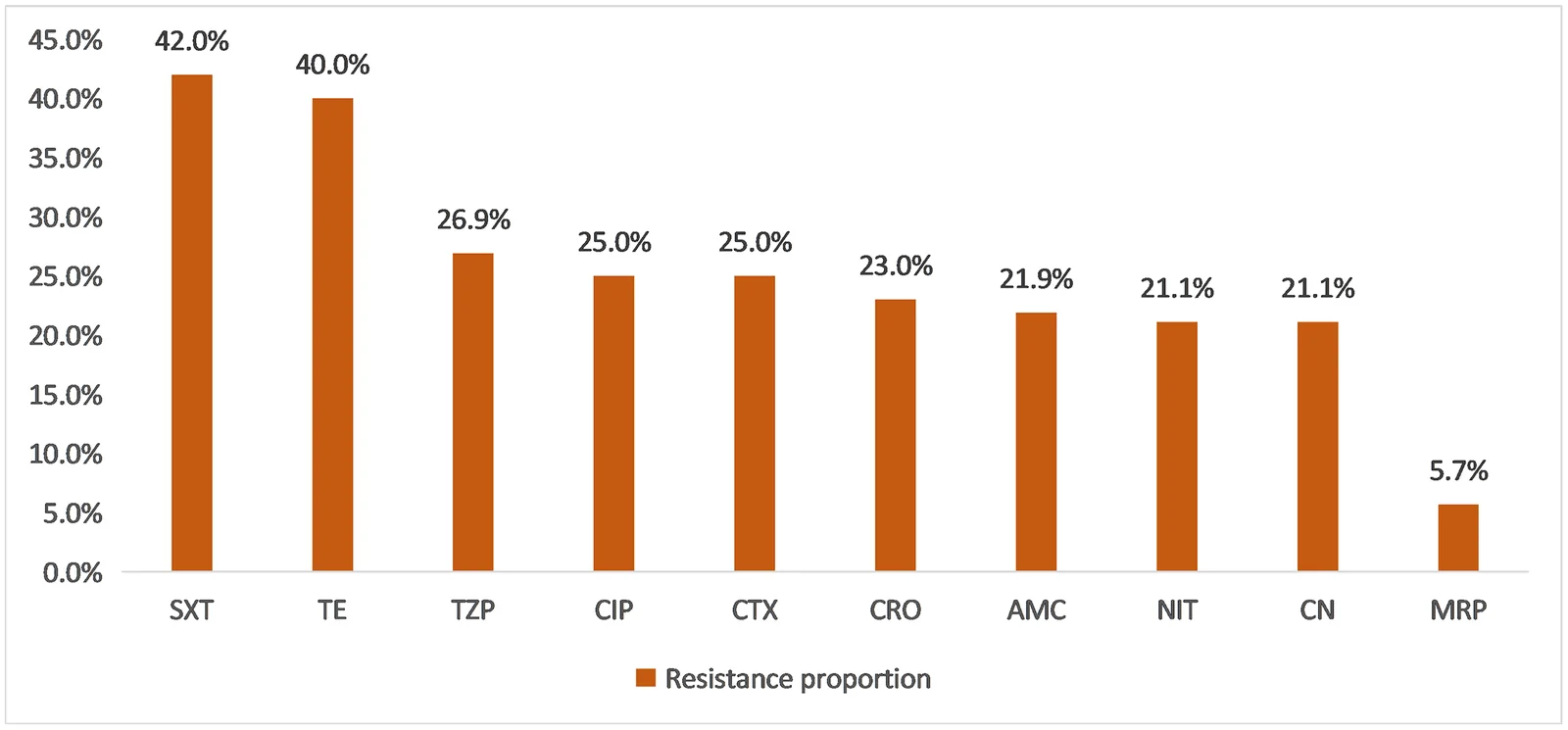
Resistance proportion of bacteria isolates against tested antibiotics.

### Fecal carriage of MDR bacteria among primary school children

A total of 109 gastrointestinal bacterial isolates were recovered from stool samples of primary school children. *Escherichia coli* was the most frequently isolated species, accounting for 87.2% (95/109) of all isolates, as shown in Fig 3. ESBL production was confirmed to be 67.6% (73/108) (ESBL testing was not applicable for *Acinetobacter* spp) of the tested Gram-negative bacteria. Additionally, multidrug resistance (MDR) was observed in 94.5% (103/109) of all isolates.

**Fig 3:**
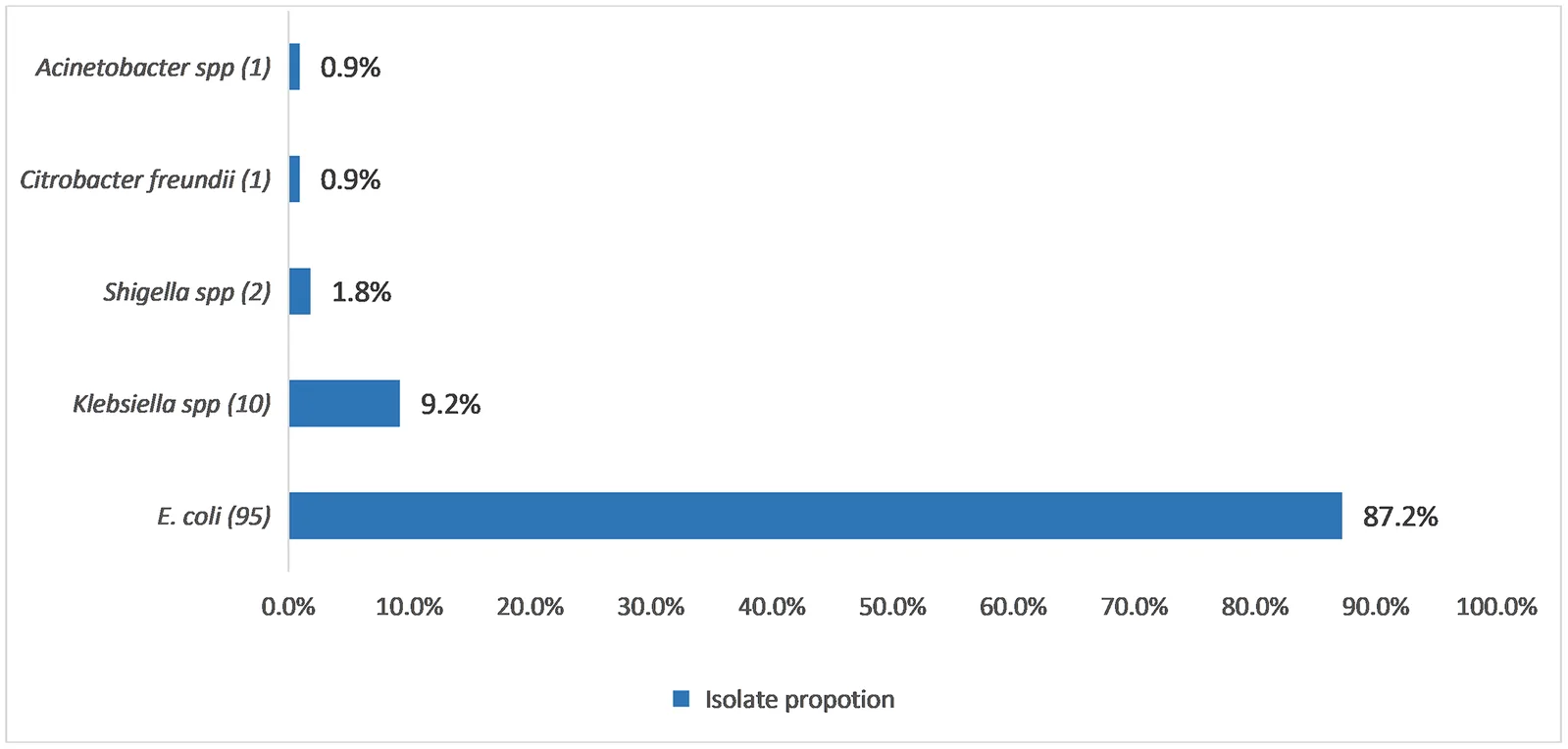
Carriage proportion of GIT bacteria in primary school children.

There was a significant difference in MDR bacteria gut carriage between UTI positive and negative children (28.3% vs 12.2%, p = 0.001).

## Discussion

This study assessed the co-infection prevalence of urogenital schistosomiasis (UGS) and urinary tract infections (UTIs) among primary school children in Meatu District, Tanzania. The observed prevalence of UGS (44.4%) was markedly higher than the 24.3% reported by Amoo *et al.* 2017 among a similar population in Nigeria [25]. This high burden of UGS reflects persistent endemic transmission and highlights the urgent need for targeted public health interventions, including sustained mass drug administration with praziquantel, health education, and improved water, sanitation, and hygiene (WASH) infrastructure to prevent reinfection and disease progression [26,27]. The higher prevalence of urogenital schistosomiasis among children in lower primary classes may reflect more frequent risky water contact and lower awareness of preventive measures. In contrast, older children may have adopted safer behaviours and benefited from repeated school-based praziquantel treatment and health education. However, because class-specific water-contact behaviours were not directly assessed, these explanations should be interpreted cautiously.

The prevalence of microbiologically confirmed bacterial UTI was found to be 12.8%, with *Escherichia coli* emerging as the predominant uropathogens. Although slightly lower than the 19.3% reported by Amoo et al. (2017) [25], these findings are concerning given the notable levels of antimicrobial resistance observed. High resistance proportion to commonly used antibiotics such as trimethoprim-sulfamethoxazole and tetracycline were documented. Moreover, over one-third of all bacterial isolates were classified as multidrug-resistant (MDR) which may pose a significant challenge to empirical treatment approaches. These findings align with similar studies in sub-Saharan Africa, where misuse of antibiotics, over-the-counter availability, and weak antimicrobial stewardship have driven the emergence and spread of resistant pathogens [28–30].

The presence of UTI and UGS co-infection was detected in 5.2% of the study population, which is lower than the 19.3% reported in the Nigerian cohort [25]. This disparity may stem from differences in demographic composition, as the Nigerian study included older female participants who are at increased risk of UTIs and UGS due to anatomical and behavioral factors [31]. The present study identified fever and frequent urination as independent predictors of UTI and urogenital schistosomiasis (UGS) co-infection among primary school children. Children with fever had significantly higher odds of co-infection, consistent with its role as a systemic response to infection, particularly in bacterial UTIs where it often reflects disease severity [32,33]. In UGS, fever may also arise from immune responses to parasite egg deposition, further contributing to the clinical presentation. Similarly, frequent urination was strongly associated with co-infection, which aligns with the overlapping urinary symptoms seen in both UTIs and *Schistosoma haematobium* infection [34,35].

Although hematuria and abdominal pain showed increased odds of co-infection, they were not statistically significant after adjustment. Hematuria, a classic feature of UGS due to bladder wall damage, may have lacked significance due to the small number of co-infected cases, a limitation also noted in similar studies [36]. In addition, the sample size was calculated using the prevalence of UGS rather than UGS–UTI co-infection because co-infection prevalence estimates were unavailable during study design. This may have limited the precision of co-infection estimates and the ability to detect weaker associations. Abdominal pain is a non-specific symptom and may be influenced by other conditions, reducing its predictive value. Sex was not associated with co-infection, suggesting similar exposure risks among male and female children, consistent with findings from endemic settings [37]. Painful urination, although excluded from the multivariate model due to perfect prediction, remains a clinically important symptom supported by its strong univariate association and existing literature [35,38]. Overall, these findings emphasize the importance of fever and urinary frequency as key clinical indicators of co-infection, highlighting the need for integrated symptom-based screening alongside laboratory diagnosis in endemic areas.

Importantly, children with heavy *Schistosoma haematobium* infection loads showed significantly higher UTI prevalence, suggesting that schistosomiasis-induced mucosal damage may facilitate bacterial colonization and subsequent infection. Schistosoma eggs deposited in the urinary tract can provoke chronic inflammation, ulceration, and squamous metaplasia, all of which compromise epithelial integrity and increase susceptibility to secondary infections [39,40]. The health implications of such co-infections are profound. Concurrent parasitic and bacterial infections may complicate clinical presentations, delay diagnosis, and adversely affect treatment outcomes [41,42]. Children suffering from UGS and UTI co-infections are at higher risk of chronic bladder pathology, hydronephrosis, and renal impairment if not managed promptly [43]. This calls for integrated diagnostic protocols that screen for both conditions simultaneously, especially in endemic areas where clinical resources are limited.

Clinically, hematuria was significantly associated with UGS, while fever and frequent urination were linked to UTI. Painful urination was common for both infections, this shared symptom likely reflects overlapping inflammatory pathways involving urothelial irritation and damage [8]. These findings support the utility of symptom-based screening algorithms for early identification of UGS and UTIs in low-resource settings where laboratory diagnostics may not be readily available [44]. However, the observed association between haematuria and UGS–UTI co-infection should be interpreted cautiously because of the wide confidence interval, indicating limited precision that warrants confirmation in larger studies.

Furthermore, the gastrointestinal tract carriage of MDR bacteria was alarmingly high, with a prevalence of 25.6%. *E. coli* was again the most common isolate, and over 90% of all stool isolates were MDR. This aligns with previous reports indicating that school-aged children in LMIC’s often serve as reservoirs for MDR bacteria due to frequent and often unregulated exposure to antibiotics, coupled with inadequate hygiene and sanitation [45–47]. Studies have shown that gut colonization with MDR organisms can serve as a precursor to subsequent endogenous infections [48]. Additionally, the potential for horizontal transmission via contaminated surfaces and poor hand hygiene in school settings further underscores the public health threat posed by these colonizing organisms [16,17].

## Conclusion & recommendation

This study demonstrates a high burden of urogenital schistosomiasis (UGS) and a notable prevalence of urinary tract infections (UTIs), with evidence of co-infection among primary school children in Meatu District, Tanzania. The findings reveal that UGS remains highly endemic, while UTIs are largely driven by *Escherichia coli*, including a concerning proportion of multidrug-resistant strains. Although the overall prevalence of co-infection was relatively low, its clinical significance is considerable, particularly given the observed association with fever and frequent urination. The results further suggest a potential synergistic interaction between UGS and UTIs, where schistosomiasis-related urinary tract damage may facilitate bacterial colonization and infection. The high levels of antimicrobial resistance and gastrointestinal carriage of multidrug-resistant organisms among children further highlights the risk of potential UTI from these reservoirs.

Based on the study findings, we recommend that integrated school-based screening programs for both UGS and UTIs be strengthened to allow early identification and management, particularly among children presenting with key symptoms such as fever and frequent urination. Given the observed antimicrobial resistance and gastrointestinal carriage of multidrug-resistant organisms, strengthening antimicrobial stewardship and regulating antibiotic use are critical to prevent further resistance development among potential UTI causing bacteria.

### Study limitation

One limitation of this study is that the sample size was based on the prevalence of urogenital schistosomiasis rather than UGS–bacterial UTI co-infection due to the lack of co-infection prevalence data from similar settings at the time of study design. This may have reduced the precision of co-infection estimates and the ability to detect weaker associations. Future studies should use emerging co-infection prevalence data to inform sample size calculations. Although haematuria was independently associated with UGS–UTI co-infection, the wide 95% confidence interval indicates limited precision; therefore, the strength of this association should be interpreted cautiously and confirmed in larger studies. Additionally, the relatively small number of co-infected children may have limited the statistical power to identify associations with less common clinical characteristics.

## Supporting information

S1 File UTI & Schistosomiasis data set. (XLSX)
